# Intelligent Neonatal Blood Perfusion Assessment System Based on Near-Infrared Spectroscopy

**DOI:** 10.1109/JTEHM.2025.3532801

**Published:** 2025-01-22

**Authors:** Hsiu-Lin Chen, Bor-Shing Lin, Chieh-Miao Chang, Hao-Wei Chung, Shu-Ting Yang, Bor-Shyh Lin

**Affiliations:** Department of PediatricsKaohsiung Medical University Hospital 89234 Kaohsiung 807 Taiwan; Department of Respiratory TherapyCollege of MedicineKaohsiung Medical University 38023 Kaohsiung 807 Taiwan; Department of Computer Science and Information EngineeringNational Taipei University 63284 New Taipei City 237 Taiwan; Institute of Imaging and Biomedical PhotonicsNational Yang Ming Chiao Tung University Tainan 300 Taiwan; College of Biological Science and TechnologyNational Yang Ming Chiao Tung University Hsinchu 300 Taiwan

**Keywords:** Neonatal intensive care unit, shock, blood perfusion, near-infrared spectroscopy, neural network

## Abstract

High-risk infants in the neonatal intensive care unit often encounter the problems with hemodynamic instability, and the poor blood circulation may cause shock or other sequelae. But the appearance of shock is not easy to be noticed in the initial stage, and most of the clinical judgments are subjectively dependent on the experienced physicians. Therefore, how to effectively evaluate the neonatal blood circulation state is important for the treatment in time. Although some instruments, such as laser Doppler flow meter, can estimate the information of blood flow, there is still lack of monitoring systems to evaluate the neonatal blood circulation directly. Based on the technique of near-infrared spectroscopy, an intelligent neonatal blood perfusion assessment system was proposed in this study, to monitor the changes of hemoglobin concentration and tissue oxygen saturation simultaneously and further estimate the neonatal blood perfusion. Several indexes were defined from the changes of hemoglobin parameters under applying and relaxing pressure to obtain the neonatal perfusion information. Moreover, the neural network-based classifier was also used to effectively classify the groups with different blood perfusion states. From the experimental results, the difference between the groups with different blood perfusion states could exactly be reflected on several defined indexes and could be effectively recognized by using the technique of neural network. Clinical and Translational Impact Statement—An intelligent neonatal blood perfusion assessment system was proposed to monitor the changes of hemoglobin concentration and tissue oxygen saturation simultaneously and further estimate the neonatal blood perfusion (Category: Preclinical Research)

## I. Introduction

Newborns usually experience the major physiological changes from inside to outside of the uterus at birth, in particular, their cardiopulmonary function. If some problems occur in this process and it results in the poor circulation of body tissues, the newborn may be life-threatening due to that the poor tissue circulation may progress to shock. The possible symptoms of shock include weakness, decreased urine amount, increased apnea, poor activity, poor digestion, cyanosis, increased oxygen demand, seizure, etc. Here, cyanosis and increased oxygen demand are the symptoms related to hypoxia and ischemia due to the lack of oxygen content in the blood. High-risk infants in the neonatal intensive care unit often suffer from hemodynamic instability. Hemodynamics is the ability of the cardiovascular system (CVS) to allow organs to function properly, the ability of blood to carry oxygen, and the ability of related tissues and organs to regulate [1]. In the previous medical studies, they indicated that the physiology and pathophysiology of the infant during this period depend on his/her behavior of blood circulation [2]. In clinical, the capillary refilling time (CRT) is the required time for the tissue color to recover when lightly pressing to whiten the nail bed and then after releasing the pressure, and it is used as a rapid indicator to measure the peripheral blood perfusion [3]. However, the result of CRT is easily affected by the subjective countdown and is difficult to measure quantitatively. Therefore, how to accurately and effectively monitor the hemodynamic function of the infant is important to alert the tissue perfusion and oxygenation insufficiency as early as possible before irreversible damage occurs [4].

Several methods, such as laser Doppler perfusion imaging (LDPI) [5] and laser Doppler flow meter (LDF) [6], may be applied in the evaluation of the blood circulation state in clinical researches. LDPI is usually applied in the non-invasive monitoring of blood flow rate. In this system, the direction of the light source illuminating into the tissue will be controlled by the computer to scan and provide the 2D image of skin capillary perfusion. However, the scan of 2D skin capillary perfusion image is time-cost, and it is not suitable for accessing the short-time change of blood flow [7]. Laser Doppler flow meter, measuring the Doppler frequency shift caused by the movement of red blood cells, is also a non-invasive measurement on the evaluation of blood flow change, i.e. the speed change of moving red blood cells [8]. This technique is limited to a qualitative index of skin blood flux rather than muscle blood volume changes, and moreover, LDF is also susceptible to the high spatial variability of capillary density and motion artifacts [9]. The technique of sidestream dark field (SDF) imaging, that is a kind of imaging mode based on stroboscopic 530 nm-wavelength LED ring, is also developed for clinical observation of vascular distribution [10]. Orthogonal polarization spectroscopy (OPS) imaging is a also non-invasive handheld device based on the polarized light technology to detect the absorption of hemoglobin, and it can be used to observe the vascular distribution of the skin at a depth of 3 mm [11], [12]. Contrast-enhanced magnetic resonance angiography (CE-MRA) is a non-invasive vascular imaging system, using the inherent magnetism of body tissues and blood in an external magnetic field to generate the vascular images, and it requires the use of the contrast agents and longer imaging time [13]. The above vascular image systems can only observe the vascular distribution on the skin surface, and cannot evaluate the state of tissue perfusion directly.

Near-infrared spectroscopy (NIRS) is a non-invasively measuring technique for monitoring the hemoglobin concentration change in the tissue [14]. In recent years, it has been widely developed in the clinical applications, such as cerebral blood oxygen [15], [16], [17], peripheral blood circulation [18], [19], [20], and neonatal brain blood oxygen monitoring [21], [22]. Therefore, the NIRS technique may contain the potential of assessing the neonatal blood perfusion information. Based on the NIRS technique, an intelligent neonatal blood perfusion assessment system was proposed to non-invasively evaluate the state of neonatal foot blood perfusion in this study. In the proposed system, a wireless blood perfusion monitoring device was designed to monitor the change of hemoglobin parameters under the applied pressure. Several indexes, extracted from the change of hemoglobin parameters, were also defined to describe the neonatal blood perfusion state in this study. Finally, the technique of neural network was also applied to classify the groups with different neonatal blood perfusion states.

## II. Methods

### A. Design of Smart Blood Perfusion Monitoring System

When incident light passes through a medium, such as human tissue, it will be attenuated due to scattering and absorption. The change of the scattering and absorption characteristics of the medium also depends on the change of light wavelength. For the wavelength range of visible light, most of human tissues are almost transparent and provide lower penetration depth due to higher scattering characteristics. For the infrared light, it contains a better penetration ability and higher sensitivity to hemoglobin and water. Therefore, the change of hemoglobin concentration can be simply estimated via the change of multi-wavelength optical intensity within the wavelength range of red and infrared light and the modified Beer-Lambert Law (MBLL) [23]. When incident infrared light passes through the human tissue, its optical attenuation $\mathrm {\Delta }OD\left ({{ \lambda }}\right)$ corresponding to the wavelength $\lambda $ due to the changes of oxy-hemoglobin ( $\mathrm {\Delta }\left [{{ {HbO}_{2} }}\right]$) and deoxy-hemoglobin ( $\mathrm {\Delta [}Hb]$) concentrations can be simply expressed by

\begin{align*} \Delta OD\left ({{ \lambda }}\right )& = -\log \frac {l_{o}\left ({{ \lambda }}\right )}{l_{i}\left ({{ \lambda }}\right )} \\ & = (\varepsilon _{{HbO}_{2}}(\lambda )\!\cdot \!\Delta \left [{{ {HbO}_{2} }}\right ]\!+\!\varepsilon _{Hb}(\lambda )\!\cdot \!\Delta \left [{{ Hb }}\right ])\!\cdot \!L\!\cdot \!B(\lambda ) \tag {1}\end{align*}

where $I_{i}\left ({{ \lambda }}\right)$ and $I_{o}\left ({{ \lambda }}\right)$ denote the optical intensities of incident light and light reflected from the human tissue respectively, and $\varepsilon _{{HbO}_{2}}\left ({{ \lambda }}\right)$, and $\varepsilon _{Hb}\mathrm {(\lambda)}$ are the molar extinction coefficients of oxy-hemoglobin ( ${HbO}_{2}$) and deoxy-hemoglobin (*Hb*) corresponding to the wavelengths $\lambda $ respectively. *L* is the distance between the light source and the photodiode. $B(\lambda)$ is a factor used to correct the actual light-traveling path [24]. Because the intersection of the absorption spectrum for ${HbO}_{2}$ and *Hb* is at about 800 nm, the 700 nm, and 910 nm infrared lights were used in this study. Then, the change of ${HbO}_{2}$ and *Hb* concentration can be obtained by the least square approximation method [25].

\begin{equation*} \left [{{ \frac {\Delta \left [{{ {HbO}_{2} }}\right ]}{\Delta [Hb]} }}\right ]=\frac {1}{L}\mathrm {\cdot }{(\mathbf {E}^{T}\mathrm {\cdot \mathbf {E})}}^{-1}\mathrm {\cdot }\mathbf {E}^{T}\mathrm {\cdot \mathbf {A}} \tag {2}\end{equation*}

where $\begin{aligned} \mathbf {E=}\left [{{\begin{array}{cccccccccccccccccccc} \varepsilon _{{HbO}_{2}}\left ({{ \lambda _{1} }}\right) & \varepsilon _{Hb}(\lambda _{1}) \\ \varepsilon _{{HbO}_{2}}\left ({{ \lambda _{2} }}\right) & \varepsilon _{Hb}(\lambda _{2}) \\ \end{array}}}\right] \end{aligned}$, and $\begin{aligned} \mathbf {A=}\left [{{\begin{array}{cccccccccccccccccccc} \frac {\Delta OD\left ({{ \lambda _{1} }}\right)}{\mathrm {B(}\lambda _{1})} \\ \frac {\Delta OD\left ({{ \lambda _{2} }}\right)}{\mathrm {B(}\lambda _{2})} \\ \end{array}}}\right] \end{aligned}$

Finally, the change of the total hemoglobin concentration ( $\mathrm {\Delta }[HbT]$) and tissue oxygen saturation ( $\mathrm {\Delta }\left [{{ {StO}_{2} }}\right]$) can be obtained from $\mathrm {\Delta }\left [{{ {HbO}_{2} }}\right]$ and $\mathrm {\Delta [}Hb]$, i. e.

\begin{align*} \Delta [HbT]& = \mathrm {\Delta }\left [{{ {HbO}_{2} }}\right ]+\mathrm {\Delta [}Hb] \tag {3}\\ \Delta \left [{{ {StO}_{2} }}\right ]& = \frac {\mathrm {\Delta }\left [{{ {HbO}_{2} }}\right ]}{\Delta \left [{{ {HbO}_{2} }}\right ]+\Delta [Hb]}\cdot 100\% \tag {4}\end{align*}

**FIGURE 1.**
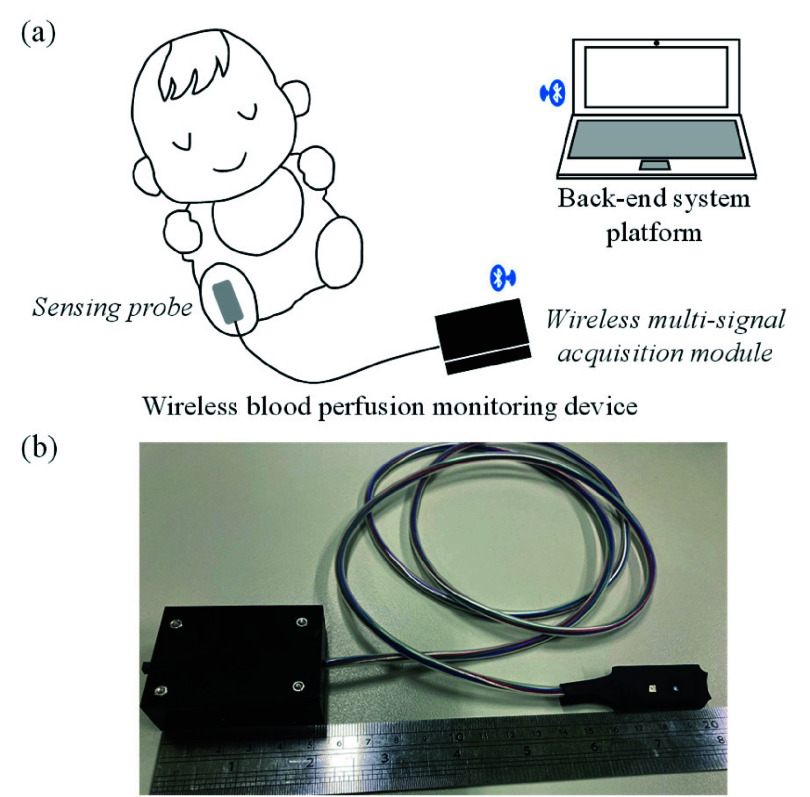
(a) Basic scheme, and (b) photographs of proposed intelligent neonatal blood perfusion assessment system.

Based on the above optical theorem, the smart blood perfusion monitoring system was designed and its basic scheme and photograph are shown in Fig. 1 (a) and Fig. 1 (b) respectively, and it mainly contained a wireless blood perfusion monitoring device and a back-end system platform. Here, the designed wireless blood perfusion monitoring device consisted of a sensing probe and a wireless multi-signal acquisition module. The sensing probe was placed on the sole of the foot, and the sensing probe would be applied with a pressure and then released repeatedly. The sensing probe would continuously monitor the change of the applied pressure signal between the sensing probe and the foot sole, and the change of the optical signal related to the blood flow. Next, the acquired pressure and optical signals would be amplified, filtered, and digitized by the wireless multi-signal acquisition module, and then would be transmitted to the back-end system platform wirelessly. Finally, the back-end system platform would analyze the received pressure and optical signals to estimate the blood perfusion state at the neonatal foot sole.

The sensing probe contained an optical blood-flow sensor and a pressure sensor, and they were placed on the front side and the back side of the sensing probe respectively. The design of the optical blood-flow sensor was based on the technique of near-infrared spectroscopy, and it mainly consisted of a dual-wavelength light-emitting diode (LED; SMT 640/700/910, Marubeni, Japan) and a photodiode (PD; PD15-22/TR8, Everlight Electronics, Taiwan). Here, the dual-wavelength LED and the PD were used to provide a dual-wavelength light source to the human tissue and receive the light reflected from the human tissue respectively. The distance between the LED and PD was set to 10 mm, and the penetration depth of the LED light is about half the LED-PD distance [26]. The pressure sensor (Flexiforce A301-1, Tekscan, USA) was used to monitor the applied pressure between the human tissue and the sensing probe.

**FIGURE 2.**
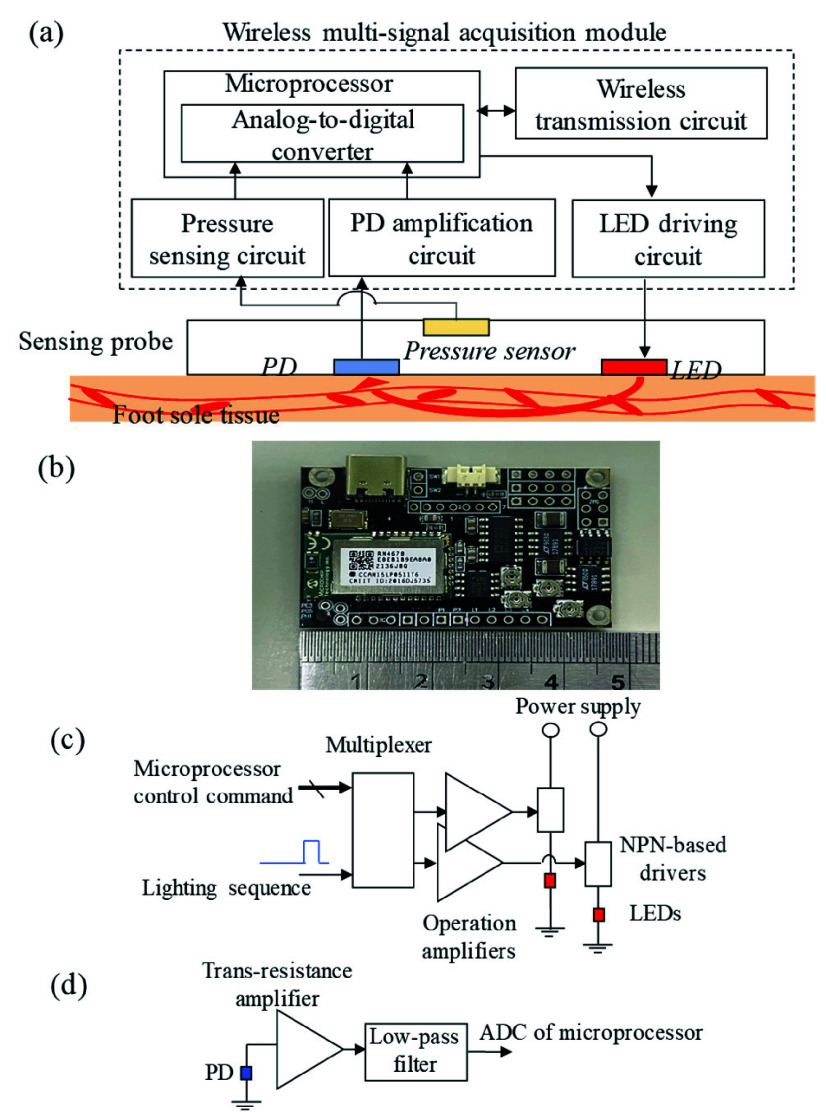
(a) Block diagram and (b) photograph of designed wireless multi-signal acquisition module, (c) LED driving circuit and (d) PD amplification circuit.

The block diagram and photograph of the designed wireless multi-signal acquisition module is shown in Fig. 2, and it mainly consisted of a LED driving circuit, a PD amplification circuit, a pressure sensing circuit, a microprocessor, and a wireless transmission circuit. Here, the LED driving circuit was designed to turn on/off the LEDs via the control command of the microprocessor, and provide a stable current to drive LEDs, and it mainly consisted of a multiplexer, operation amplifiers, and NPN transistors. The multiplexer was used as the LED selector to turn on/off the connection between the operation amplifiers and NPN transistors. The operation amplifier was used to provide a stable voltage on the emitter of the NPN transistor to allow a steady current passing through these LED. The design of PD amplification circuit was based on a 20M trans-resistance amplifier with a 1.5k Hz low-pass filter and was deigned to convert the PD current into the voltage signal. The design of pressure sensing circuit was based on an instrumentation amplifier with unit gain, and was designed to monitor the change of the pressure applied in the skin of the infant.

The wireless transmission circuit was designed to transmit data to the external device wirelessly, and it complied the Bluetooth 4.0 specification. In the beginning, the LED driving circuit would first be controlled by the microprocessor to turn on the dual-wavelength LED to illuminate the human tissue. Next, the reflected light intensity would be received by the PD on the sensing probe, and then would be amplified by the PD amplification circuit and digitized by the analog-to-digital converter of the microprocessor. Finally, the data of the reflected light would be sent to the wireless transmission circuit to transmit to the back-end system platform wirelessly.

**FIGURE 3.**
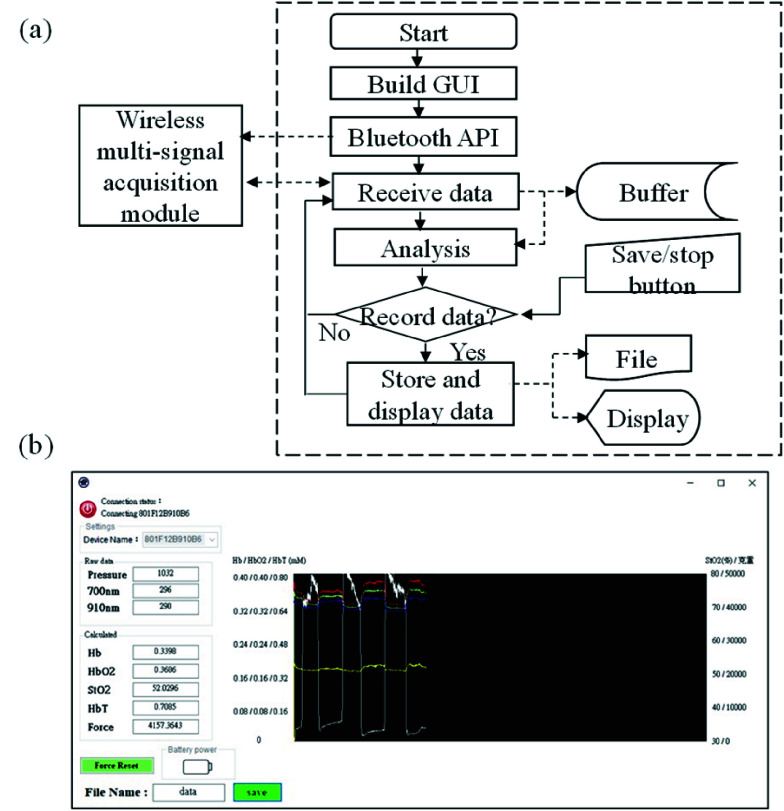
(a) Flowchart and (b) screenshot of multi-signal monitoring program built in back-end system platform.

The design of the back-end system platform was based on a commercial laptop with the operation system of Windows 10. In this system platform, a multi-signal monitoring program was also developed to receive, display, store and analyze the bio-signals from the wireless multi-signal acquisition module. The flowchart and the screenshot of the developed monitoring program are shown in Fig. 3 (a) and Fig. 3 (b) respectively.

### B. Experimental Design

The experiment was performed Kaohsiung Medical University Chung-Ho Memorial Hospital, Kaohsiung, Taiwan. A total of 102 newborns participate, and all of their parents agreed and signed the informed consent form (No. of institutional review board is KMUHIRB-SV(I)-20200004). In clinical, the premature infant, that his/her mean blood pressure is smaller than the gestational age (GA), is often considered to contain higher risk of the poor blood circulation and may require the further treatment [27], [28]. The mean blood pressure is one of the important organ perfusion indicators, calculated by combining 1/3 of systolic blood pressure with 2/3 of diastolic blood pressure [29]. Therefore, according to the premature infant and mean blood pressure, the newborns were divided into four groups in this experiment. Here, Group I (17 males and 7 females): full-term infant ( $\ge 37$ weeks) and the mean blood pressure was higher or equal to his/her GA. Group II (11 males and 3 females): full-term infant and the mean blood pressure was lower than his/her GA. Group III (29 males and 22 females): premature infant (<37 weeks) and the mean blood pressure was higher or equal to his/her GA. Group IV (5 males and 8 females): premature infant and the mean blood pressure was lower than his/her GA. The averaged postnatal age was 2.3±1.4 days. The information of participating infants is summarized in Table 1.

**TABLE 1:** Information of Infants in Different Groups

Group	Group I	Group II	Group III	Group IV
Total No.	24	14	51	13
Postnatal age (day)	2.7±1.6	2.1±1.7	2.3±1.2	1.5±0.9
Male No.	17	11	29	5
Female No.	7	3	22	8
GA week (week)	> 37	> 37	< 37	< 37
Mean blood pressure	> GA week	< GA week	> GA week	< GA week

**FIGURE 4.**
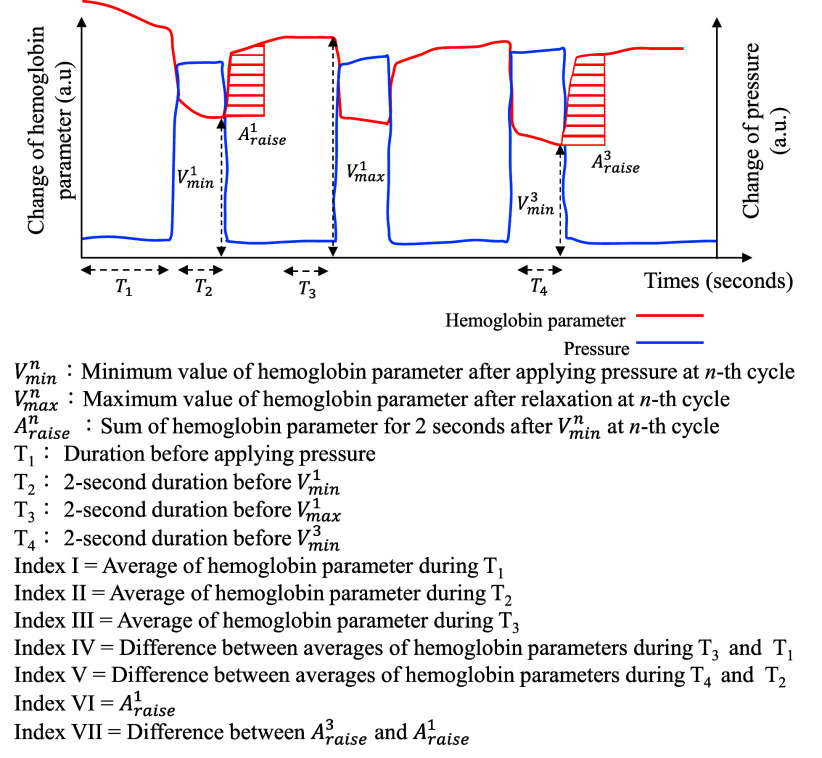
Illustration for definition of indexes related to neonatal blood perfusion state.

The sensing probe would be placed on the foot sole of the newborn first. The medical staff would apply a pressure on the sensing probe for 5 s and then release the applied pressure for 10 s. The above procedure would be repeated 3 times. Each participant would be measured for six times within one hour. According to the changes of HbT and ${\mathrm {StO}}_{2}$ under this experiment, several parameters related to the neonatal blood perfusion are first defined and illustrated in Fig. 4. Here, Index I was designed to monitor the initial blood circulation state of the tissue. Index II was designed to monitor the blood perfusion state of the tissue under applying pressure. Index III was designed to monitor the blood perfusion recovery state of the tissue after applying pressure. Index IV was designed to monitor the difference between the initial blood perfusion state and the recovered blood perfusion state after applying pressure. Index V was designed to monitor the difference between the blood perfusion states under first time pressure and repeated pressure. Index VI was designed to monitor the blood perfusion rate of the tissue. Index VII was designed to monitor the difference between the blood perfusion rates after applying first time pressure and repeated pressure. The t-test method was used to analyze the significant difference, and the significance is defined as $p < 0.05$. Kruskal-Wallis test (nonparametric test) was also performed to examine whether there exist significant differences among groups.

### C. Neonatal Blood Perfusion Classification

**FIGURE 5.**
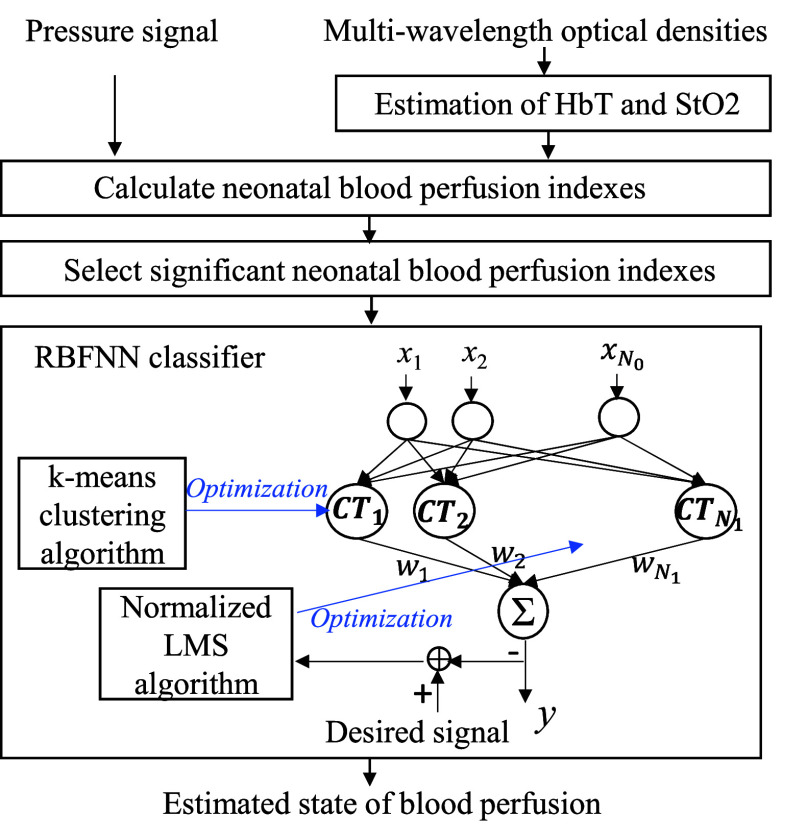
Flowchart of neonatal blood perfusion classification.

In order to classify the blood perfusion status of newborns, a radial basis function neural networks (RBFNN), containing the advantages of simple three-layer feedforward network configuration, fast training process, requirement of lower training data, and excellent non-linear approximation ability, was used in this study [30], [31]. The flowchart of neonatal blood perfusion classification is shown in Fig. 5. The neonatal blood perfusion indexes would first be obtained from the pressure signal, HbT and StO2. Next, the significant neonatal blood perfusion indexes would be selected as the input vector of RBFNN classifier. Under $N_{0}$ input, $N_{1}$ hidden, and one output neurons, the RBFNN output $y\left ({{ n }}\right)$ can be obtained by

\begin{equation*} y\left ({{ n }}\right )={\boldsymbol {\Phi }}^{T}\left ({{ n }}\right )\cdot \boldsymbol {w} \tag {5}\end{equation*}

where $\boldsymbol {w}={[w_{1},w_{2}\mathrm {, \cdots ,}w_{N_{1}}]}^{T}$ is the weight vector, and $w_{\mathrm {k}}$ denotes the weight between the *k*-th hidden neuron and the output neuron. ${\boldsymbol {\Phi }}\left ({{ n }}\right)={[\phi _{1}\left ({{ n }}\right),\phi _{2}\left ({{ n }}\right)\mathrm {, \cdots ,}\phi _{N_{1}}(n\mathrm {)] }}^{T}$ is the output vector of the hidden neurons [33], [34], and the output of the *k*-th hidden neuron can be expressed by the Gaussian basis function [32]

\begin{equation*} \phi _{k}\left ({{ n }}\right )=\exp \left ({{ -\frac {\left \|{{ \boldsymbol {x}\left ({{n }}\right )-{\boldsymbol {CT}}_{\boldsymbol {k}} }}\right \|^{2}}{2\sigma ^{2}}}}\right ) \tag {6}\end{equation*}

where ${\boldsymbol {CT}}_{\boldsymbol {k}}$ is the center vector in the *k*-th hidden neuron, *x* $\left ({{ n }}\right)={[x_{1}\left ({{ n }}\right),x_{2}\left ({{ n }}\right), \cdots ,x_{N_{0}}(n\mathrm {)] }}^{T}$ is the *n*-th input dataset vector and $\sigma ^{2}$ is the estimated variation of input datasets. Here, $\left \|{{.}}\right \|$ is the calculation of the Euclidean distance between the two vectors. Here, the center is used to represent the representative prototype of the data sample in the feature space, and it also determines the discriminative power. Here, the k-means clustering algorithm [35] and the normalized least mean square (LMS) algorithm [36] were used to train the hidden neuron center vectors and the weight vector respectively.

In the training stage, the desired outputs of RBFNN were defined as 0 and 1 corresponding to the good blood perfusion group (Group I) and poor blood perfusion group (Group IV) respectively. The learning model of the RBFNN is a fuzzy output between 0 and 1. Therefore, in order to determine the blood perfusion state, the RBFNN threshold has to be defined in the training stage. When the RBFNN output was greater than the given threshold, it would be classified to the poor blood perfusion group, and otherwise, if the output value was smaller than the given threshold, it would be classified to the good blood perfusion group. Finally, the accuracy of the RBFNN model would be tested and confirmed to validate the classification performance in the testing stage, after the learning of the RBFNN model was completed.

## III. Results

### A. Neonatal Blood Perfusion Indexes Corresponding to Different Groups

**FIGURE 6.**
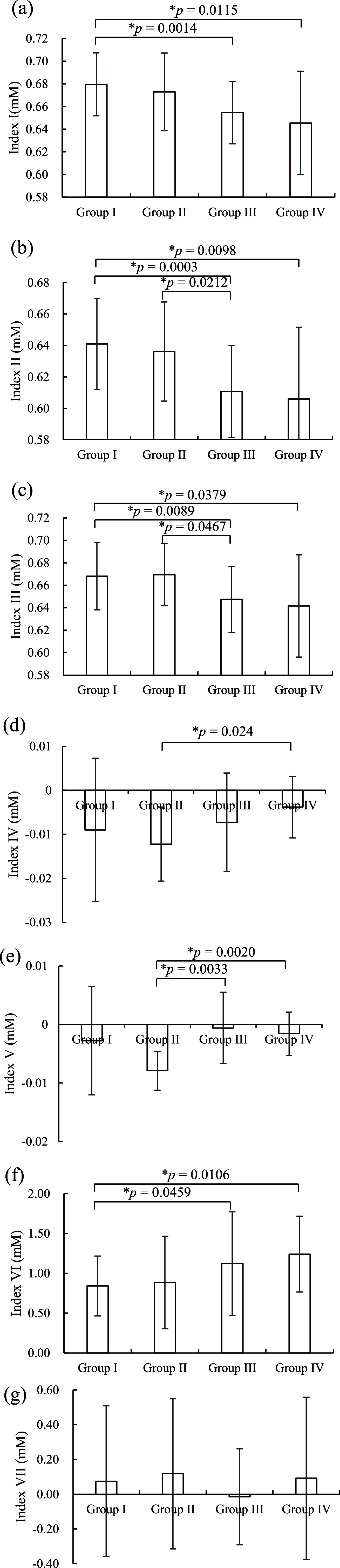
(a) Index I, (b) Index II, (c) Index III, (d) Index IV, (e) Index V, (f) Index VI, and (g) Index VII of total hemoglobin corresponding to different groups.

In this section, the defined indexes related to the state of the neonatal blood perfusion corresponding to different groups were first investigated. Fig. 6 shows the means and standard deviations of indexes obtained from the estimated total hemoglobin concentration corresponding to different groups. Here, the total hemoglobin concentration is positively proportional to the hemoperfusion volume in the local tissue. Compared with the factor of the blood pressure, the influence of the gestational age factor on the Index I-III of HbT was more significant. Index I-III values of HbT for two full-term infant groups (Group I and Group II) were higher that of two premature infant groups (Group III and Group IV). Here, the Index I-III values of means and standard deviations ( $0.6795 \; \pm \; 0.0278$; $0.6409 \; \pm \; 0.0289$; $0.6680 \; \pm \; 0.0300$) of HbT for the Group I were significantly higher than that of the Group III ( $0.6546 \; \pm \; 0.0275$, $p \; = 0.0014$; $0.6108 \; \pm \; 0.0293$, $p \; = 0.0003$; $0.6475 \; \pm \; 0.0295$, $p \; = 0.0089$) and that of the Group IV ( $0.6454 \; \pm \; 0.0456$, $p \; = 0.0115$; $0.6060 \; \pm \; 0.0455$, $p \; = 0.0098$; $0.6416 \; \pm \; 0.0455$, $p \; = 0.0379$) respectively. The Index II- III values ( $0.6361 \; \pm \; 0.0315$; $0.6695 \; \pm \; 0.0276$) of HbT for the Group II were also significantly higher than that of the Group III ( $p \; = 0.0212$; $p \; = 0.0467$) respectively. Moreover, Index VI values of HbT for two full-term infant groups were lower than that of two premature infant groups. Here, the Index VI value of HbT for the group I ( $0.8399 \; \pm \; 0.3756$) was significantly lower than that of the Group III ( $1.1222 \; \pm \; 0.6491$, $p \; = 0.0459$) and that of the Group IV ( $1.2393 \; \pm \; 0.4759$, $p \; = 0.0106$) respectively.

**FIGURE 7.**
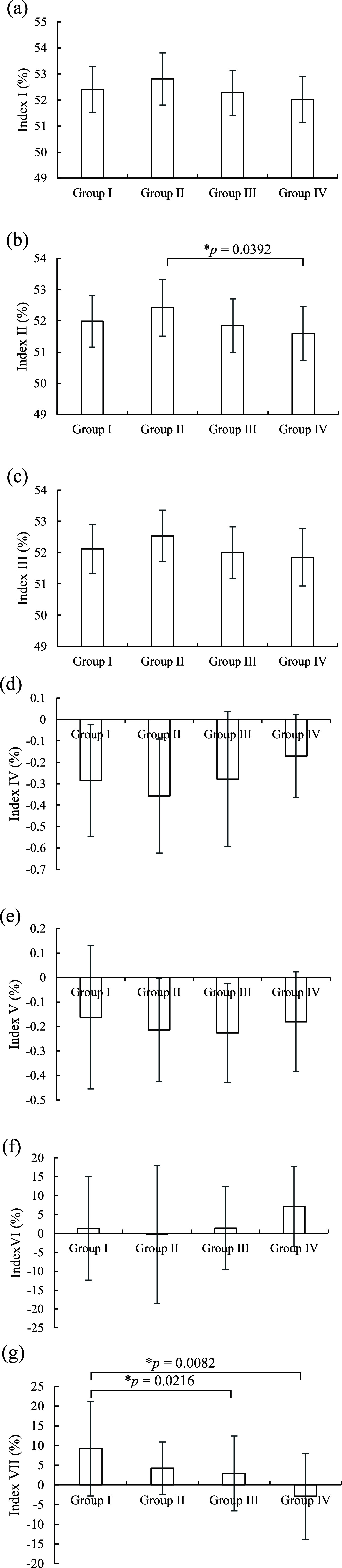
(a) Index I, (b) Index II, (c) Index III, (d) Index IV, (e) Index V, (f) Index VI, and (g) Index VII of tissue oxygen saturation corresponding different groups.

Fig. 7 shows the means and standard deviations of indexes obtained from the estimated tissue oxygen saturation corresponding to different groups. The influence of the gestational age and blood pressure factors on all index value changes of ${\mathrm {StO}}_{2}$ seemed insignificant. But the Index VII value of ${\mathrm {StO}}_{2}$ for Group I ( $9.2185 \; \pm \; 12.0192$) was significantly higher than that of the Group III ( $2.9323 \; \pm \; 9.5071$, $p \; = 0.0216$) and Group IV ( $2.8658 \; \pm \; 10.8890$, $p \; = 0.0082$). The Index II value of ${\mathrm {StO}}_{2}$ for Group II ( $52.4154 \; \pm \; 0.9043$) was also significantly higher than that of the Group IV ( $51.5932 \; \pm \; 0.8693$, $p \; = 0.0392$).

### B. Performance Evaluation of Blood Perfusion Assessment System

According to the experimental results in the previous section, there was a difference in blood perfusion between Group I and Group IV in Index I-III and Index VI of HbT and Index VII of ${\mathrm {StO}}_{2}$. Therefore, these indexes were used as the input features of neural networks to estimate the blood perfusion state. Before test, the optimal parameters of the neural network have to be determined first in the training stage. Here, the number of hidden neurons was set to 16, 32, and 64, and the threshold was set between 0 and 1. In order to evaluate the performance of the proposed system, the parameters of the binary classification test are defined as followings: The parameters of the binary classification test are defined here: True Positive (TP): the good blood perfusion group is correctly recognized as the good blood perfusion group; False Positive (FP): the good blood perfusion group is incorrectly recognized as the poor blood perfusion group; True Negative (TN): the poor blood perfusion group is correctly recognized as the poor blood perfusion group; False Negative (FN): the poor blood perfusion group is incorrectly recognized as the good blood perfusion group. Here, F-measure and accuracy are used to determine the optimal parameters of neural network. F-measure is the harmonic average of sensitivity and positive predictive value (PPV), and can be calculated as followings.

\begin{equation*} \mathrm {F-measure=2\cdot }\frac {sensitivity\cdot PPV}{sensitivity+PPV} \tag {7}\end{equation*}

where the definitions of sensitivity and PPV are $\frac {TP}{TP+TN}$ and $\frac {TP}{TP+FP}$ respectively

\begin{equation*} \mathrm {Accuracy}=\frac {TP+TN}{TP+FP+TN+FN} \tag {8}\end{equation*}

Here, a total of 70 trials randomly selected from the Group I and Group IV were used for training. When the hidden neuron number and the threshold were set to 64 and 0.5 respectively, the better classification performance can be obtained (PPV = 91.43%, sensitivity = 91.43%, F-measure = 91.43%, accuracy = 91.43%).

**FIGURE 8.**
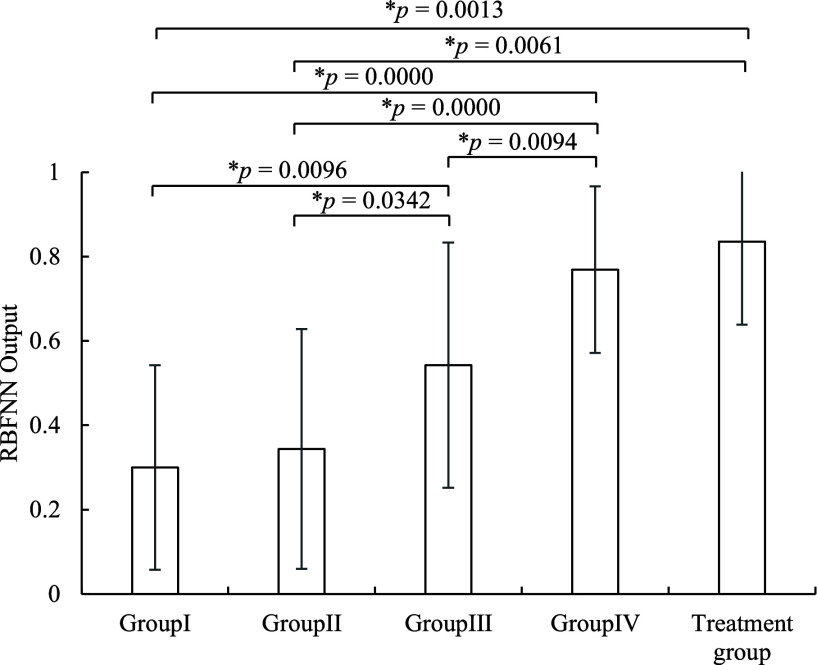
RBF output values corresponding of different groups.

Next, a total of 30 trials selected from the Group I and Group IV were used for test, and the hidden neuron number and the threshold were also set to 64 and 0.5 respectively. It shows the classification performance for test was also good (PPV = 81.25%, sensitivity = 86.67%, F-measure = 83.87%, accuracy = 83.33%). The outputs of RBFNN corresponding to different groups are shown in Fig. 8. As our expectation, the output value of RBFNN would increase from the Group I to Group IV. Here, the RBFNN output of the Group IV ( $0.7687 \; \pm \; 0.1969$) was significantly higher than that of Group I ( $0.2997 \; \pm \; 0.2426$, $p \; = 0.0000$), Group II ( $0.3438 \; \pm \; 0.2844$, $p \; = 0.0000$) and Group III ( $0.5426 \; \pm \; 0.2906$, $p \; = 0.0094$). The RBFNN output value of Group III was also higher than that of Group I ( $p \; = 0.0096$) and Group II ( $p \; = 0.0342$). The average output value of RBFNN for the newborns who need to be treated from the physician evaluation was 0.8349.

## IV. Discussions

From the experimental results, the Index I values of HbT for two full-term infant groups were higher than that of the two premature infant groups. Because the HbT concentration is related to the blood volume in the tissue, it reflected that the general blood volume in the tissue of the premature infant might be lower and it might be explained by that the relatively lower blood vessel density and the larger proportion of small blood vessel in premature infants [37]. Therefore, it might also cause the relatively severe vascular occlusion or small vessels state in the tissue of the premature infant, when applying a pressure on the infant skin. The results of that Index II values of HbT for two premature infant groups were relatively lower, also fitted the above phenomenon. Moreover, the Index III values for two full-term infant groups was higher than that of the two premature infant groups. Here, the Index III values was related to the perfusion volume in the tissue after releasing the pressure. Besides the difference between the blood vessel density of full-term and premature infants, it also might be affected by the relatively small cardiac output in premature infants [38], [39]. However, the Index VI values, related to the perfusion rate after releasing the pressure, for two full-term infant groups was relatively lower. For the full-term infant groups, the difference between the blood volumes under the conditions of applying and relaxing the pressure was relatively small, and it also caused the relatively lower perfusion rate after relaxing the pressure. Index IV, V, and VII were related to the change of the perfusion volume, vascular occlusion, and perfusion rate after applying the repeated pressures. Although several significant differences between the Index IV/V/VII of different groups, but the relationship between these Index values and the factors of gestational age or blood pressure seemed unobvious.

For most of the ${\mathrm {StO}}_{2}$-based indexes, the relationship between these index values and the factors of gestational age or blood pressure also seemed unobvious. The above phenomenon might be explained by that the short-term vascular occlusion state caused by the pressure affected the oxygen consumption slightly. However, the difference between the Index VII values of ${\mathrm {StO}}_{2}$ for the full-term infant group with the blood pressure higher than his/her gestational age, and the premature infant group with the blood pressure lower than his/her gestational age was significant. Here, the Index VII was related to the change in the recovery state of blood oxygen saturation concentration after applying the repeated pressure. The blood volume and cardiac output of premature infants were generally lower to cause the supply of lower fresh blood. Therefore, the blood oxygen supply of premature infants was relatively insufficient to result in a relatively slow recovery rate of blood oxygen after continuous squeezing. Moreover, the use of RBFNN classifier with the input of these significant parameters exactly could provide a good performance of estimating blood perfusion state (PPV = 91.43%, sensitivity = 91.43%, accuracy = 91.43%). The output value of RBFNN also increased with the gestational age and blood pressure of the infant, and it might be a good index related to the blood perfusion state.

The comparison of the proposed system and other systems for assessing the blood circulation state in clinical is summarized in Table 2. The methods of LDPI and LDF are based on the noninvasive Doppler frequency shift techniques to monitor the information of the blood flow change. But, LDPI is a scanning image method that the longer scanning image time is required, and cannot obtain the information of short-term blood flow [40], and the measuring depth of blood flow rate estimation is sallow (about 1 mm) and is easily affected by the change of the tissue contour and scanning distance [41], [42], [43]. The spatial resolution of LDF is relatively poor, and it is not suitable to distinguish the depth information of blood vessels [44]. Moreover, both of LDPI and LDF only monitor the changes of the blood flow in the local tissue, and cannot distinguish the newborns blood perfusion state directly. The SDF and OPS imaging techniques are the vascular imaging systems. The techniques of SDF and OPS imaging also require the longer scanning image time and the experienced operators [45]. Here, OPS provide the lower image resolution to cause the underestimation of blood flow [10], [46], and both of SDF and OPS are easily susceptible to pressure artifacts [47]. CE-MRA is a vascular imaging technology, providing the clear spatial distribution of blood vessels. However, the contrast agent is required in CE-MRA, and it may cause harm to newborns [13]. Different from the above methods that only provide information of the local blood vascular distribution or blood flow rate, the proposed system could non-invasively and real-time monitor the changes of hemoglobin concentration and tissue oxygen saturation simultaneously. The experimental results also showed some defined indexes exactly contain significant differences between different groups under the procedure of applying and relaxing a pressure on the tissue, and different groups could be effectively discriminated via the technique of neural network with these significant indexes. Compared other deep learning approaches, we are not sure that the use of RBFNN is the best classifier for this research. The use of other excellent linear or non-linear classifiers with significant neonatal blood perfusion indexes should also provide a good classification performance. But the RBFNN advantages of fast training process, requirement of fewer training data, and excellent non-linear approximation ability are obviously suitable for this research. Moreover, the sensing probe has to be firmly in contact with the skin surface to avoid influence of environmental light on hemoglobin measurement. The proposed system also contained the advantages of non-invasive and real-time measurement, low cost, and simple operation procedure. Although the proposed system lacked of detail blood vessel spatial information, it might be used in the clinical application in the future to help doctors assess the blood perfusion status of newborns.

**TABLE 2:** Comparison Between Proposed System and Other Systems

	Proposed system	LDPI [5]	LDF [6]	SDF imaging [10]	OPS imaging [11]	CE-MRA [13]
Measurement technique	Near-infrared spectroscopy	Doppler frequency shift	Doppler frequency shift	Sidestream dark field technology	Polarized light technology	Nuclear magnetic resonance
Wavelength of light source	700, 910 nm	670 nm	670 or 780 nm	530 nm	548 nm	-
Portable device	Yes	No	No	Yes	Yes	No
Analysis parameters	Hemoglobin parameters	2D blood flow image	Blood flow velocity	2D blood vessel image	2D blood vessel image	3D angiography
Classifier	RBFNN	-	-	-	-	-
Applications	Estimation of peripheral blood perfusion	Estimation of peripheral vascular disease and diabetic conditions	Estimation of tissue or skin micro-circulation blood flow	Capillary imaging on organs surface	Observation of sublingual micro-circulation	Assess vascular disease around organs
Advantages	Simultaneous measurement of changes in blood oxygen and blood perfusion	Spatial information of blood flow, non-contact measurement	Higher time-resolution in measurement	Visualized blood spatial distribution information	Visualized blood spatial distribution information	Spatial distribution information of blood vessels, non-contact measurement
Limitations	Lack of detail blood vessel spatial information, influence of sensing probe contact tightness	Influence of tissue contour and scanning distance on measurement, longer scanning time, shallow measuring depth	Influence of motion artifacts, only relatively local blood flow information	Longer scanning time, influence of pressure artifact	Lower imaging resolution, longer scanning time, influence of pressure artifact	Expensive cost, requirement of contrast agent

## V. Conclusion

In this study, an intelligent neonatal blood perfusion assessment system was proposed to estimate the neonatal blood perfusion. The basic concept in this study is to monitor the changes of hemoglobin concentration and tissue oxygen saturation simultaneously by using the technique of NIRS under applying and relaxing pressure to indirectly evaluate the neonatal blood perfusion. Here, according to the changes of HbT and ${\mathrm {StO}}_{2}$ with pressure, several indexes related to neonatal blood perfusion were also proposed. From the experimental results, the influence of the gestational age factor on the blood neonatal circulation of newborns seemed more obvious than the blood pressure factor. The averaged hemoglobin concentration in the tissue of the premature infants was significantly lower, and it was also more easily affected when applying a pressure on the tissue. But after relaxing the pressure, the averaged perfusion volume and rate of the full-term infants seemed lower than that of the premature infants. Moreover, for the premature infants, the recovery speed of the blood oxygen saturation after the repeated pressure was relatively slower. Finally, by using the technique of neural network, the proposed system could effectively distinguish different groups from these significant indexes. The proposed system also contained the advantages of that non-invasive and real-time measurement, simple operation procedure and lower cost. Therefore, the proposed system may contain the potential of assisting the physicians in evaluating the neonatal blood circulation state in the future.
